# The Sargasso Sea

**Published:** 1881-07

**Authors:** 


### THE SARGASSO SEA.

This is the name given to a portion of the Atlantic Ocean covered with the seaweed, sargassum. Its boundaries may be indicated by tracing a triangle, of which the three corners are represented by the Azores, the Canaries and Cape de Verde. Within those limits the sea is clothed on its surface with a garment of vegetable material, so thick as to retard the progress of vessels through it. Steamers avoid it, because of the fouling of their screws and paddles by the weed ; but sailing vessels from Europe to the West Indies, South America, the Cape of Good Hope, etc., must push through it. When Columbus, on his first voyage, had got some distance to the westward of the Canary Islands, he was amazed to find his ships in what looked like a meadow. As far as he could see, the water was covered with a greenish-yellow plant, as water-lilies cover a pond. This was the first time such a thing had been seen, and the sailors were scared. Columbus could not explain the sight he saw, and might have thought with his men that the weed was the covering of some dangerous rock which lay a short distance down, ready to tear and rend them. The lead was hove, but no bottom was found. The ships kept on their course, and in a few days they got clear of the weed.

